# Rapid Recovery after Reparation of Full-Thickness Chondral Defects of the Knee with the Use of Hyaluronan (HA)-Based 3-D Scaffold

**DOI:** 10.3390/jfb14100491

**Published:** 2023-09-24

**Authors:** Nicolas Valladares, Monica Araceli Cabrero Montes, Gibran J. Jacobo-Jimenez, Maria G. Zavala-Cerna

**Affiliations:** 1 División of Sports Medicine, Rehabilitem, Guadalajara 44100, Mexico; nvalladares@hotmail.com (N.V.); dra.aracelicabrero@gmail.com (M.A.C.M.); dr.gibranjacobo@gmail.com (G.J.J.-J.); 2 Laboratorio de Investigación en Inmunología, Unidad Académica Ciencias de la Salud, Universidad Autónoma de Guadalajara, Zapopan 45129, Mexico

**Keywords:** scaffold-based, microfractures, chondral defects, knee surgery, surgical approaches

## Abstract

Articular cartilage injuries are found in up to 60% of patients who undergo an arthroscopic knee procedure, and those that totally affect articular cartilage (grade IV) have limited regenerative capacity and extended time for recovery. 3-D scaffolds represent a novel solution to address this type of injury. Our purpose was to analyze the MRI findings and functional status of patients that underwent repair of chondral defects either by microfractures or Hyaluronan (HA) 3-D scaffolding. We conducted a retrospective study of patients with chondral defects. The outcomes analyzed in this study included anatomical changes evaluated by the Henderson score (based on MRI findings) at baseline, 6, and 12 months after surgery, and improvement in functionality evaluated by the Modified Cincinnati Knee Rating System (MCKRS) at baseline and 6 months after surgery. Clinical and demographic characteristics were similar for both groups. There was a statistically significant improvement in Henderson score for the 3-D scaffold-treated group at 6 months versus the microfracture group (*p* < 0.0001). Improvement in functionality, measured by the MCKRS, was more frequently found in the 3-D scaffold-treated group. In conclusion, the use of HA 3-D scaffolding was superior, with faster recovery evident 6 months after the surgery that progressed to full recovery in all patients a year after surgery. Future studies with a randomized design might help to support our findings. This study provides level III evidence.

## 1. Introduction

Lesions of the knee cartilage are very frequent, and it is estimated that they occur in up to 60% of patients that, for any reason, undergo a knee arthroscopic procedure [1]. Patients with these lesions frequently experience pain, swelling, functional impairment, and a reduction in the quality of life [2]. There is an inadequate capacity for self-repair of chondral defects due to the limited regenerative and healing properties of the hyaline cartilage, secondary to low vascularization and innervation; it has been documented frequently that untreated lesions can predispose the joint to increased cartilage loss and early-onset osteoarthritis (OA) [3,4].

Chondral lesions are most commonly found on the patella and medial femoral condyle [5]. Lesions that include chondral full-thickness are classified as grade IV according to the International Cartilage Repair Society (ICRS) and are considered a real challenge for treatment, often with a slow or difficult-to-achieve full recovery [2,3]. Several treatment options for symptomatic patients have been developed; however, no clear gold-standard treatment has been established since most of the interventions depend on several factors, such as the size and place of the lesion, the presence of osteoarthritis and other comorbidities, and additional demographic variables [6].

Currently, the treatment of cartilage defects is a clinical challenge, not just from the point of view of the surgery and the technique implemented but also because, after the surgical intervention, the resulting reparative fibrocartilage has inferior biomechanical properties when compared to the native cartilage, and even more, reparative fibrocartilage seems to be more susceptible to degenerative changes leading to early-onset OA [7].

Several surgical techniques have been proposed to try to improve the regeneration of the articular cartilage. They can be broadly divided into bone marrow stimulation with microfractures (MF), cartilage replacement, and cartilage regeneration [8]. 

MF is considered first-line surgery and is composed of a single-stage arthroscopic procedure where the penetration of the subchondral bone induces the release of mesenchymal stem cells (MSCs) to promote cartilage repair [9]. For cartilage replacement and cartilage regeneration, a variety of cell-based approaches have been developed, including two-step autologous chondrocyte implantation (ACI) [10,11] or MSC direct implantation [12]. There are some limitations to ACI related to the need for two interventions: the first to harvest cells from synovial fluid and the second to implant the in vitro transformed cells into chondrocytes, making this a rather expensive and lengthy intervention [11]. Other problems related to both are the lack of cell adherence, fixation, and retention; therefore, there is uncertainty about defect repair by the implanted cells. The design and use of scaffolds have allowed for proper cell implantation without the need to further damage the chondral surface [13]. Scaffolds allow for better cellular fixation and retention. However, there are some characteristics that scaffolds should meet in order to properly contribute to the healing process: they must (a) possess the requisite material properties to promote neocartilage formation, (b) exhibit sufficient mechanical integrity for handling during implantation and afterwards for joint mobility, and (c) be reliably fixed within cartilage during surgery [14].

Enrichment of scaffolds with hyaluronic acid (HA) has been associated with good clinical results in a case report of a patient with a large chondral lesion treated with microfractures and a cell-free polymer-based cartilage implant that contained polyglycolic acid and HA [15], as well as for chondrocyte implantation [16].

HA, officially identified as hyaluronan, is a natural polymer composed of unsulfated glycosaminoglycan with a high molecular weight, formed from the repetitive accumulation of molecular chains of N-acetyl-glucosamine and glucuronic acid [17]. Several studies have found that when injected intraarticularly for OA, due to its physical properties, it promotes joint lubrication and improves shock absorbency during movement to enhance synovial fluid viscosity, which has been associated with benefits in comparison to placebos for pain and function and improvements in patient global assessment scores with no negative side effects [18]. 

Previous studies have demonstrated that HA promotes the formation of new hyaline-like cartilage tissue due to an upregulation in chondrocyte proliferation and differentiation, as well as contributing to an inhibition of the enzymatic cartilage degradation that normally happens in inflammatory conditions [19]. In the presence of an inflammatory condition of the knee, it is estimated that the concentration of HA is reduced by 33–50% and therefore can have satisfactory clinical results, but at an elevated cost since they will need repeated interventions [17]. 

Despite these benefits, there is still an ongoing debate on whether surgical treatment with MF is superior to scaffold-associated procedures for the treatment of large chondral defects, primarily due to a lack of standardization in techniques, differences in study designs, and poor outcome definitions, rendering the use of this new approach limited [8]. Additionally, there is a wide array of scaffold designs, and therefore there is a need to elucidate the optimal scaffold option for future human trials to advocate for homogeneity in terms of exposures.

The aim of this study was to analyze functionality and radiographic recovery in patients that had surgical intervention for the reparation of full-thickness cartilage lesions (ICRS grade IV) of the knee, either with a HA-based 3-D scaffold or the microfracture technique, over a one-year period. 

## 2. Materials and Methods

### 2.1. Study Population

This was a retrospective study where the participants were patients who fulfilled the following inclusion criteria: >18 years old, with a full-thickness cartilage lesion (ICRS grade IV) of the knee, who underwent arthroscopy in the same center, and had the same surgeon during the observation period January–December of 2019. The exclusion criteria were the requirement for bone grafting, osteonecrosis, and the presence of concomitant inflammatory arthropathies that required intra-articular injections of steroids.

Patients were divided into two groups according to the technique used during the arthroscopic intervention: microfractures (MF) and HA 3-D scaffold groups. The decision for the technique selected was based on the availability of the patients’ insurance company to pay for the HA 3-D scaffold.

Clinical information from patients was obtained from the electronic medical records. Baseline assessments were obtained from measurements performed on the day of admission for surgery. At this point, we collected information related to demographic aspects and the characteristics of the lesion. After the surgery, we searched for patients’ clinical information at 6 and 12 months.

### 2.2. Ethical Considerations

The study was reported to the local IRB, and a signature of informed consent was exempt due to the retrospective nature of the study (CEI/2023/001).

### 2.3. Surgical Techniques

Surgery was performed on all patients under spinal anesthesia with routine sterile preparation and draping. After arthroscopic evaluation, the knee was approached with a mini-arthrotomy, and the chondral defect was prepared and debrided with the use of curettes to remove chondral-free fragments and calcified lesions while avoiding penetration of the subchondral bone. Damaged cartilage was removed until the defect was evident, and approximately 5 mL of bone marrow aspirate cells (BMACs) were harvested from the femur. The aspirate was then located into the patient’s lesion, either by the generation of microfractures with BMA directed through the cylindric containment of the fracture or with the use of a three-dimensional scaffold (Hyalofast^®^ Anika Therapeutics Inc., Bedford, MA, USA), which is composed of a single 3-D hydrophilic, fibrous layer of benzyl ester of hyaluronic acid (HA) imbibed in the BMACs. For the latter, chondral defects were measured vertically and horizontally, then the three-dimensional scaffolds were tailored to the defect size and shape of the lesion, soaked in BMAC, and implanted in the defect site. The scaffolds were secured to the surrounding previously cleaned cartilage using intraarticular sutures or an arthroscopic retracting system.

### 2.4. Functional Status

To evaluate the patient’s functional status, the Modified Cincinnati Knee Rating System (MCKRS) was obtained preoperatively and post-surgery at 6 months.

The system consists of 12 scored questions that cover the domains of pain, swelling, function, and activity level. The total score is calculated as the sum of all question responses, with 100 representing the best/excellent knee function and 0 representing the worst/poor knee function [20]. Results are interpreted as follows: < 30 poor, 30–54 fair, 55–79 good, and > 80 excellent.

### 2.5. MRI Assessment and Henderson Scale

Magnetic resonance imaging (MRI) results were collected preoperatively, at 6 and 12 months after the surgery, performed by an independent radiology consultant blinded to the type of cartilage repair. After the evaluation of the images, the radiologist obtained a score for each patient based on a system developed by Henderson et al. [21] Parameters reviewed included defect fill, signal, effusion, and subchondral edema, which were scored on a scale from 1 to 4, where 1 represents a normal knee and 4 represents a severe full-thickness defect.

### 2.6. Second-Look Arthroscopy

In some cases, a second-look arthroscopy was performed due to the presence of mechanical symptoms, crepitus, or pain, and for those who required surgical treatments for other reasons unrelated to the previous surgical procedure. During the second-look arthroscopy, grafts were inspected and evaluated according to the ICRS cartilage repair assessment scoring system, which includes the degree of defect, fill, graft integration to the adjacent normal articular surface, and gross appearance of the graft surface.

### 2.7. Statistical Methods

Descriptive analysis was performed by mean and SD for continuous data and proportions for categorical data; as non-parametric alternates, we used medians and interquartile ranges. Inferences were made by using the chi-square for categorical data, the Student’s *t*-test (two-tailed) for continuous data, and the Kruskal-Wallis for variables with a non-parametric distribution. We conducted an analysis of response profiles to test if there was a difference in the pattern of change over time in patients receiving the two interventions. Significant differences were set at a value of *p* < 0.05. We used the statistical software STATA IC 16.

## 3. Results

We included a total of 33 patients who underwent surgery during the period from February to December 2019. The group treated with HA 3-D scaffold was composed of 12 patients, and the group treated with microfractures was composed of 21 patients. All patients had lesions classified as grade IV according to the ICRS; their functional status was of fair performance, and only a few had poor performance status. Most lesions were located on the right knee, and 90% of patients reported having an amateur type of physical activity. Table 1 shows frequencies and comparisons for both groups without significant distinctions at baseline.

With respect to surgery and the immediate postoperative period, there were no significant complications reported for any patient. Six months after the surgery, patients were asked to undergo a new MRI to assess changes in their Henderson scale score. A statistically significant improvement was found in the group treated with the 3-D scaffold to a median (ICR) of 1 (1–1.5) (*p* < 0.001), which was not evident in the microfracture-treated group.

**Figure 1:**
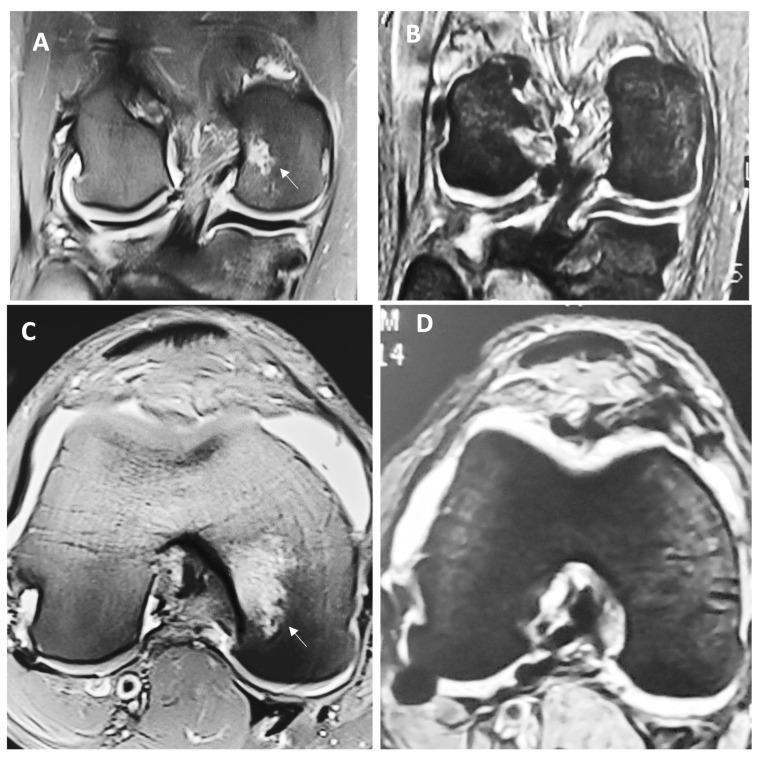
Representative MRI view of the full-thickness cartilage defect and adjacent tissue before (**A**) and (**C**) and 6 months after the surgery (**B**) and (**D**) in a patient treated with HA 3-D scaffold. Letter (**A**) represents the anteroposterior view of the knee, while letter (**C**) demonstrates the coronal view; both are showing cartilage damage with ICRS grade IV in the medial femoral condyle (arrow), with effusion and evident subchondral edema (Henderson score of 4). Letters (**B**) and (**D**) represent the same respective views 6 months after the intervention, where the lesion has visibly disappeared and reparative tissue is layering above; additionally, effusions and edema are absent (Henderson score of 1).

Figure 1 depicts macroscopic changes observed at 6 months in the MRI. Then, 12 months after the surgery, there was a continued improvement for both groups, but this difference was markedly important for individuals in the HA 3-D scaffold group, since all patients from this group were found to have a Henderson scale score of 1, while the group treated with microfractures still had almost half of the individuals (47.6%) with a score of 2 (*p* = 0.0048). This means that all patients treated with the HA 3-D scaffold returned to a radiographic image that represents a knee with an absence of fluid and edema in the joint. Conversely, in the microfractures group, this was not achieved by any patient. Table 2 represents these comparisons between groups.

With respect to functionality, patients in the group treated with HA 3-D scaffold were able to progress to excellent functionality (>80) in the following 6 months, having a near-perfect score in the MCKRS, which represents the capacity to perform physical activity, while in the group treated with microfractures, the highest score was related to only a moderate capacity for physical activity.

**Figure 2:**
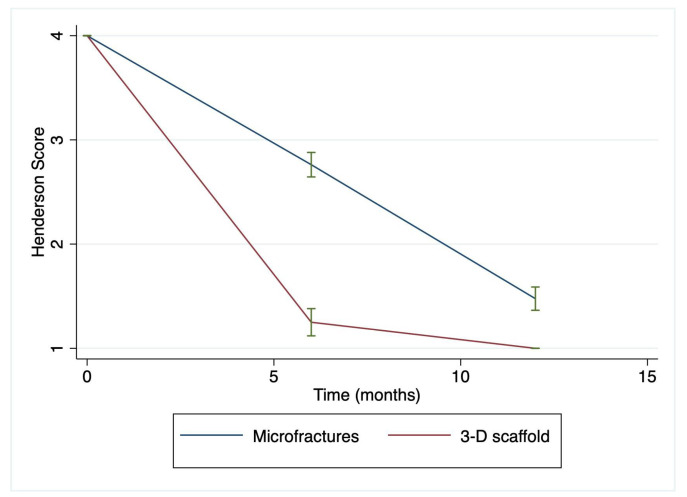
Henderson score over time in the microfractures- and HA 3-D scaffold-treated groups. Parameters reviewed for this score included defect fill, signal, effusion, and subchondral edema, which were scored on a scale from 1 to 4, where 1 represents a normal knee and 4 represents a severe full-thickness defect. Of notice, there is a significantly faster recovery at 6 months in the HA 3-D scaffold-treated group compared to the MF group, which continued 12 months after the surgery when all subjects within the 3-D scaffold group achieved a Henderson score of 1. b = −1.2 (95% CI: −1.08–−3.05) and *p* < 0.0001. Green lines represent mean and SD.

**Figure 3:**
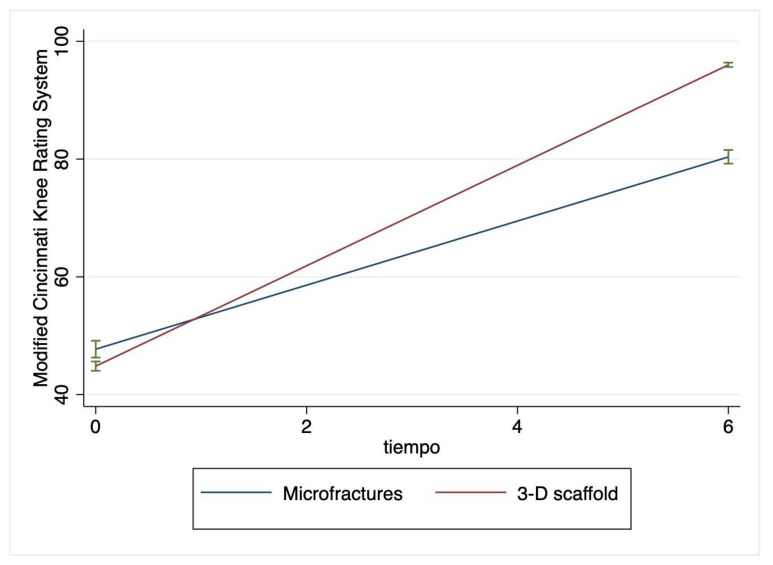
Modified Cincinnati Knee Rating System (MCKRS) score for the microfractures- and HA 3-D scaffold-treated groups. The MCKRS is composed of 12 questions related to pain, swelling, function, and activity level. The results are interpreted as follows: <30 poor, 30–54 fair, 55–79 good, and >80 excellent. There is a significant improvement at 6 months in patients in the HA 3-D scaffold group compared to the MF group. b = 32.66 (95% CI: 30.44–34.89) and *p* < 0.0001. Green lines represent mean and SD.

The mean Henderson and MCKRS scores over time can be observed to change at different rates for patients being treated with two surgical approaches (Figure 2 and Figure 3).

**Figure 4:**
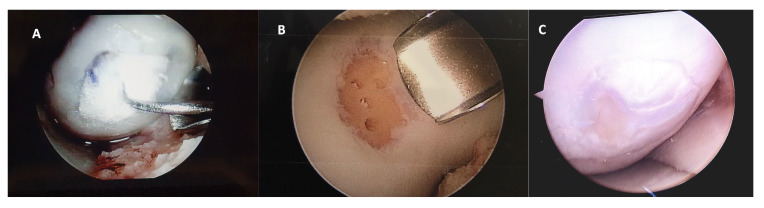
Representative macroscopic view of the cartilaginous repair tissue within the circular full-thickness cartilage defects and adjacent cartilage during surgery for (**A**) the HA 3-D scaffold and (**B**) the microfractures groups. Letter (**C**) is the macroscopic view of the second-look arthroscopy (after 12 months) for the patient treated with HA 3-D scaffold with the graft completely integrated to the normal adjacent articular surface, without evident filling defects.

After 12 months of follow-up, a small number of patients required a new second-look arthroscopy, during which the integrity of the graft was verified to assess the degree of defect, fill, graft integration to the adjacent normal articular surface, and gross appearance of the graft surface. We were unable to observe a case without integration into adjacent normal articular tissue (Figure 4).

## 4. Discussion

Our findings support an evident improvement in the group of patients treated with the use of HA 3-D scaffold, seen at 6 months after the surgery, that progressed towards the end of the study at 12 months. According to our findings, the usage of HA 3-D scaffold for the treatment of cartilage defects is effective and superior to MF, with evidence related to less time for total recovery of functional status (MCKRS) and faster improvement in MRI findings (Henderson score). Recently, similar findings about satisfactory clinical outcomes with the use of HA 3-D scaffolds were reported [22,23]. However, there is still a need to clarify its superiority when compared to MF, convey the type of scaffold with the best performance, and apply similar clinical outcomes. The use of scores based on imaging techniques such as the Henderson score could be very useful and prevent biases, especially when using instruments that rely solely on patients’ perspectives. 

Frequently, larger lesions, such as the one’s presented in the current study, will require cell-based therapies to repair the extension of the defect, due to the poor capacity of cartilage for self-repair, by either MF’s induced bone marrow stimulation, cartilage cell replacement, and/or the promotion of cartilage regeneration [24]. A matrix-induced implantation of grafted cells with the use of a scaffold could favor cartilage cell replacement, and depending on additional components of the scaffold, it could also favor the promotion of cellular regeneration [25].

A previous study with a randomized controlled design found that an aragonite-based scaffold was superior to MF in patients with grade III or superior osteochondral lesions [26]. They found that up to 78% of patients treated with the scaffold had a significant improvement compared to 34% in the MF group (*p* < 0.0001) after two years of follow-up [26]; our study showed an even faster response visible six months after surgery. A faster response and recovery observed in our study could be due to the use of HA-based 3-D scaffolds. The microstructure, porosity, and addition of supplements to the scaffold have been identified as important characteristics to allow cell fixation, proliferation, differentiation, and further invasion, leading to vascularization and tissue repair. The microstructure needs to provide the required structural strength to support external loads, and the porosity needs to allow the diffusion of nutrients and soluble factors effortlessly through the extent of the scaffold [27].

Furthermore, HA adds important characteristics to the scaffold that could explain the rapid recovery observed in our patients, such as frictionless movement of the knee and the promotion of cellular proliferation [28]. HA enhances synovial fluid viscosity and creates a hydrated pathway by which cells can move and migrate [29]. Additionally, HA increases chondrocyte proliferation and differentiation and contributes to the inhibition of enzymatic cartilage destruction [19,30]. HA can also interact with cells and growth factors that are required for cellular proliferation by binding to cell surface receptors, such as CD44 and the receptor for hyaluronan-mediated motility (RHAMM), that are expressed on osteoblasts, osteoclasts, and MSCs [31], as well as a group of growth factors, such as bone morphogenetic proteins (BMPs) and the cytokine TGF-b, important for cellular regeneration [32]. Another important characteristic of HA is its biodegradability, due to the presence of hyaluronidases in the human body, which makes this material able to be broken down and metabolized by the body when it is no longer needed [33]. Finally, it has been demonstrated in vitro that high molecular weight HA better supports neoangiogenesis, likely through a unique ability to induce clustering of CD44 receptors on endothelial cells, which is crucial for cellular proliferation [34].

With respect to cells used for cartilage replacement, osteochondral transplantation has been frequently used to approach lesions larger than 2 cm, or grade III and above. The graft can be obtained from a donor (allograft) or from the patient (autograft); in the present study, we were able to perform autografts from the patient’s femur bone marrow aspirate to imbibe the scaffold, which significantly simplified the procedure since there was no need to perform matching [35]. Additionally, there are no side effects related to the rejection of the graft, which contained high levels of mesenchymal stem cells (MSCs), cytokines, and chemokines (TGF-b and PDGF) [36]. MSCs have the capacity to differentiate into multiple lineages of mesoderm, including chondrocytes, and hold an immunomodulatory potential [37] that could also partially explain the fast recovery observed in our patients. 

The most frequent complication in surgeries associated with failure of the intervention is colonization with bacteria and the development of an infectious disease since some infectious agents can be multidrug resistant, and they are more commonly found in hospitals. The use of supplements with antibacterial properties could also aim to improve results from surgical interventions [38]. HA can be easily modified to introduce functional groups that can be used for cross-linking the incorporation of other molecules to influence surrounding tissues [39]. This could be achieved by either physical entrapment, covalent immobilization, or affinity-based interactions [40]. To further improve the observed performance of HA 3-D scaffolds for the reparation of large chondral lesions, the incorporation of antibacterial peptides could also aid in the prevention of complications, allowing an even larger number of patients to have satisfactory outcomes after surgery.

Some of the limitations in our study are related to the retrospective design, and selection bias could be present. The access to the scaffold by insurance companies might select patients to have other common attributable variables that could partially explain a faster recovery, such as increased access to additional resources for clinical improvement, including physiotherapy [41], access to new anti-inflammatory drugs, or being able to rest for a longer period of time without the need to return early to work or daily activities [42]. Other factors that could bias our results in a similar way are related to preoperative demographic and psychological variables. Even though we did analyze general demographic variables, we did not ask about income and education, nor did we evaluate psychosocial factors, which have been related to improvement in functional outcomes after knee surgery in the past [6]. New studies with an RCT design could resolve these biases.

## 5. Conclusions

In our study, the use of HA-based 3-D scaffolds proved to be superior in the postoperative period, with evidence of total recovery in radiography (Henderson scale) and faster functionality (MCKRS) when compared to the MF technique. Future studies with an RCT design would be useful to address selection bias and to learn if this benefit persists over a longer period, especially when subjects normalize their physical activity, demand for labor, and performance of physical activity.

## Figures and Tables

**Table 1:** Clinical and demographic characteristics of patients treated with microfractures or 3-D scaffolds.

Variable	Microfractures (*n* = 21)	HA 3-D Scaffold (*n* = 12)	*p*
Age (mean ± SD)	34.8 ± 5.1	32.3 ± 6.6	0.2360
Feminine gender *n* (%)	16 (76)	9 (75)	0.939
Lesion in the right knee *n* (%)	16 (76)	7 (58)	0.283
Injury of the medial condyle *n* (%)	16 (76)	7 (58)	0.283
Lesion site (condyle femoral medial) *n* (%)	16 (76)	7 (58)	0.283
Lesion size (mm) median (ICR)	125 (100–225)	100 (80–100)	0.088
MCKRS scores before surgery median (ICR)	46 (44–56)	44 (43–46)	0.2696
Henderson scale before surgery median (ICR)	4 (4–4)	4 (4–4)	0
Amateur physical activity *n* (%)	19 (91)	11 (92)	0.693

**Table 2:** Changes in the MCKRS and Henderson scores after surgery (6–12 months).

Treatment	Baseline	6 Months	*p*	12 Months	*p*
*Henderson Score*					
Microfractures	4 (4–4)	3 (2–3)	0.0001	1 (1–2)	0.0048
3-D scaffold	4 (4–4)	1 (1–1.5)		1 (1–1)	
*Modified Cincinnati Knee Rating System*					
Microfractures	46 (44–56)	81 (77–83)	0.0001	-	-
3-D scaffold	44 (43–46)	96.5 (95–97)		-	

Results presented represent median (min-max).

## Data Availability

No additional reports have been made available for the public.
